# A Complexity‐Informed Phenomenologic Study of Canadian Academic Nursing Leadership: Leading Through the Pandemic With Lessons Learned for Managing Future Crises

**DOI:** 10.1155/jonm/5076028

**Published:** 2026-10-01

**Authors:** Lillie Lum, Liza L. S. Choi

**Affiliations:** ^1^ School of Nursing York University Toronto Ontario M3J 1P3 Canada yorku.ca; ^2^ School of Nursing Mount Royal University Calgary Alberta T3E 6K6 Canada mtroyal.ca

**Keywords:** complexity nursing leadership, COVID-19 pandemic, crisis management, university nursing education

## Abstract

**Background:**

The leadership challenges in the Canadian healthcare system became more complex during the global pandemic. Major disruptions in nursing education programs occurred simultaneously, creating an acute adaptive crisis. Academic nursing leaders were required to respond to rapidly changing public health directives and institutional policies while ensuring the safety and continuity of their nursing program.

**Aim:**

The purpose of this qualitative study was to explore higher education nursing leadership experiences, including leader styles, interactions, risk management, decision‐making, changes to education programs, daily operations, and team dynamics.

**Methods:**

The study used an interpretative phenomenologically informed design, which was informed by complexity leadership theory. Semistructured, in‐depth interviews were conducted with 20 academic nursing leaders across Canada. Recruitment and data collection occurred from spring 2023 to fall 2024 during the final phases of the COVID pandemic. Interviews were recorded, transcribed, and analyzed following the principles of content analysis.

**Results:**

Content analysis of the 20 interviews revealed three main themes: *maintaining quality nursing education programs; administrative decision challenges; and communication during a crisis.*

**Conclusions:**

Study findings provide insights into the often invisible work of academic nursing leaders navigating the intersection of educational continuity, regulatory requirements, student progression, faculty workload, clinical placement disruption, institutional policy, and public health directives. Ensuring effective organizational adaptation was the major focus of complexity nursing leadership. In preparation for potential future crises, succession planning and preparation of future leaders are a priority.

## 1. Introduction

With high mortality rates and the lack of experience with a novel virus, many countries faced major challenges to their healthcare services. Disruptions in nursing education programs occurred simultaneously, creating a complex organizational crisis. While planning and implementing major changes, faculty and students were required to rapidly adapt to new ways of teaching and learning [1, 2]. Academic nursing leaders needed to respond to rapidly changing public health directives, institutional policies, clinical placement disruptions, faculty workload concerns, student progression needs, and professional regulatory expectations. These interdependent pressures required leadership practices that were adaptive, relational, and emergent rather than linear or fully predetermined. The recent pandemic highlighted the importance of effective leadership in managing crises that have the potential to create major disruptions to an organization and society [3].

Nursing education programs, under noncrisis conditions, have well‐established routines, which were suddenly disrupted by major facility lockdowns, safety concerns, and an overall lack of operational pandemic planning by governments and universities [4]. It was imperative to balance student safety and to meet program goals [5]. Yet these programs were expected to graduate students with the same level of clinical capabilities as students in noncrisis periods [6]. Immense pressure on nursing faculty and leaders came from the awareness that decisions made during the crisis had long‐term consequences and could result in harm to students and patients [7, 8]. Even universities that had a continuity of operations plan for disasters did not have plans tailored to the specific needs of nursing programs.

Academic administrators continuously face unexpected minor emergencies and operational challenges. The exceptional global impact of the pandemic, however, was a crisis that heightened the level of risk over an unknown period of time, with the potential to have long‐term destructive effects [9]. It caused unexpected consequences, threatened the safety and physical and psychological well‐being, and the operation of all educational institutions [10]. Agith et al. [3] and Uhl‐Bien et al. [11] concluded that conventional approaches to leadership are unlikely to be effective in crisis conditions. A complexity leadership perspective that enhances the overall function of an organization, leading to successful adaptation, is required.

In this study, a crisis is understood as a disruptive event or condition that creates significant uncertainty, threatens organizational functioning, and requires urgent decision‐making under conditions of incomplete information [12]. The COVID‐19 pandemic was not only an acute crisis but also a prolonged and evolving crisis that required academic leaders to repeatedly adapt programs, policies, communication, and support systems over time.

Although crises take many different forms, common features have been defined as an “unexpected, significant, disruptive, harmful or threatening event for organizations and their stakeholders leading to experiences of confusion, uncertainty and suffering” ([13], p. 154). Unexpected crises are characterized by confusion, uncertainty, and stress [14]. Conventional or generic responses to organizational disruption are inadequate. Prolonged operational disruptions and reputational damage may occur [9]. As a result, effective COVID crisis leadership required all leaders to rapidly adapt to new and unique challenges. Al‐Dabbagh [12] and Agith et al. [3] recommend that adaptive complexity leadership using a behavioral approach to reduce complexity and chaos is essential.

Universities, however, are dynamic, complex organizations with multiple layers of bureaucracy designed to maintain control and stable functioning, which often makes it difficult to implement rapid changes [15, 16]. Moerschell & Novak [9] describe universities as organizations with a high degree of complex structures, human activities, and services like small cities. A certain degree of operational unpredictability and uncertainty coexists creating a high degree of adaptive complexity [10]. These features are also characteristic of healthcare systems. Both types of organizations were mutually impacted by the pandemic. COVID‐19 provided an ideal context to examine the relevance of complexity leadership theory to better understand the relationship between leadership behaviors, social systems, and future outcomes [17]. Successful leadership results from both leaders and followers cocreating adaptive responses that produce new organizational effectiveness instead of the status quo [14]. The crisis components of a pandemic can become the catalyst for new ways of organizational functioning.

Crises can be acute or prolonged. Nevertheless, their management includes actions taken to contain, limit, and mitigate adverse events. Effective leaders foster collaboration, promote transparent communication, and enhance teamwork [3]. Moercschell & Nowak [9] recommend that leaders need to develop dynamic plans for emergencies that contain precrisis elements, crisis procedures, and postcrisis abilities to keep their staff focused and engaged. Since the pandemic created a longer‐than‐expected crisis for most organizations, leaders continued to face novel challenges. Al‐Dabbagh [12] emphasized the importance of decision‐makers taking multiple measures to ensure that all concerned understand the nature of the problem and develop a shared vision of how to address it, thereby creating voluntary consensus. Successful leaders are those who have “the ability to be visible, adaptive, empathetic, and creative and who empower others to think and act creatively” during all phases of a crisis ([16], p. 231).

Literature exploring academic nursing leaders’ role in managing major crisis situations is relatively sparse [4]. Fowler & Robbins [18] recommend that further phenomenological studies be conducted to provide an understanding of the lived experience. The primary research objective of this study was to explore how Canadian academic nursing leaders addressed the challenges of sustaining their academic programs and relationships with health service organizations during major pandemic disruptions. Exploring their experiences as strategic decision‐makers with responsibility for university degree nursing programs from an adaptive, complexity lens contributes new knowledge for future crisis preparedness.

The specific focus on academic nursing education programs does not preclude learning from the experiences of leaders within this context. Nursing leadership in clinical settings with the organizational mandate of providing patient care may be different. Yet the decision‐making processes required to sustain complex operations and human resource management necessitate complexity leadership.

Although several years have passed, this interval provided the opportunity for critical reflection about successes, failures, and the implementation of strategies to promote future adaptability of Canadian nursing education programs. The objective was to achieve a better understanding of leadership strategies supporting classroom and clinical teaching while optimizing student learning, interprofessional relations with faculty and staff, and key stakeholders.

## 2. Materials and Methods

This study was informed by phenomenological principles and complexity leadership theory to explore how academic nursing leaders made meaning of their leadership experiences during the COVID‐19 pandemic. Rather than treating leadership as a fixed set of individual traits or actions, this study conceptualized leadership as situated, relational, and emergent within complex academic, professional, regulatory, and healthcare system contexts.

A phenomenologically informed approach was appropriate because the study sought to understand participants’ lived experiences of sustaining nursing education during a period of significant uncertainty, disruption, and adaptation. The objective of this study was not to produce a universal essence of pandemic leadership, but to interpret the meanings participants attributed to their experiences as academic nursing leaders. This orientation allowed us to attend to how participants described uncertainty, decision‐making, relational responsibility, ethical tensions, and adaptation while leading nursing programs during a prolonged crisis.

The focus was on uncovering the essence of higher education nursing leadership during the pandemic, delving into leader styles, interactions, crisis management, decision‐making, changes to education programs, daily operations, and team dynamics. Complexity leadership theory provided a sensitizing framework for examining leadership as relational and emergent within dynamic systems. This was particularly relevant to academic nursing leadership during COVID‐19 because participants were not leading in isolation; they were navigating interconnected academic, clinical, institutional, and regulatory systems.

This qualitative study was prepared in accordance with the Consolidated Criteria for Reporting Qualitative Research (COREQ) guidelines, where applicable, supporting rigorous and transparent reporting of the qualitative interview design, data collection, analysis, and findings. Given the limited literature on Canadian academic nursing leadership during the COVID‐19 pandemic and the exploratory nature of the study, purposeful sampling was used to capture deep, detailed insights from a specialized group of participants. Data collection and analysis continued until thematic saturation was reached during the final stage of theme development.

### 2.1. Sampling and Recruitment

For the purposes of this study, academic nurse leaders represented senior administration who managed nursing education program activities in universities as either directors or deans. Leadership activities, as described by Fowler & Robbins [18] were directed toward achieving positive faculty and learner outcomes.

Five provinces, representing Western, Central, and Eastern Canada, were selected. Although all provinces offer nursing education programs, the provinces with the greatest number of university degree programs were approached to promote greater homogeneity of the sample. This distribution reflected the scope of recruitment responses rather than an intentional exclusion of other provinces or other types of nursing programs. Recruitment was designed to include academic nursing leaders across Canada; however, participation depended on leaders’ availability, willingness to participate, and response to recruitment invitations during a period of ongoing pandemic‐related workload pressures.

Since the study focus was on English‐speaking programs, French‐speaking programs were not approached. Criteria for recruitment included those who held a senior level of responsibility for undergraduate nursing programs for the duration of the pandemic. Although some had additional responsibility for graduate programs, the focus of the interview was solely on the undergraduate level. The research project was approved by the Research Ethics Committee of the University (Approval #e2023‐049).

Recruitment was conducted using a snowball sampling process. National and provincial nursing educator networks assisted in disseminating information about the study. A review of university nursing programs across Canada was conducted to identify potential participants and create a sample for the selected provinces. Individual recruitment emails were also sent to 55 academic nursing leaders in higher education institutions across Canada with Bachelor of Science in Nursing (BSN) and Bachelor of Nursing (BN) programs. Inclusion criteria consisted of deans or directors of nursing undergraduate degree programs in Canadian universities with major administrative responsibility during the pandemic. Administrators of diploma degree programs in colleges were excluded.

The sample consisted of 20 participants with representatives from each region.

### 2.2. Data Collection and Analysis

All participants provided voluntary written consent. Data collection was conducted from spring 2023 to fall 2024 remotely using video conferencing software. The researchers conducted and recorded semistructured interviews (virtual) over 60–90 min, capturing nuanced reflections and interpretations of interviewees’ experiences during the pandemic. No compensation for participation was offered. (Table 1).

**TABLE 1:** Interview guide.

Introduction: Thank you for your willingness to participate in this nursing leadership study. This session will be recorded. Any direct quotes will be anonymously cited. Your voluntary participation has been recorded with the signed consent.
Interview questions:
1. Years in professional practice, years in academia, years in current role
2. Please provide a brief description of your nursing program, ie. Undergraduate, graduate, size of enrollment, curricular focus, etc.
3. During the pandemic, between 2000 and 2022, describe your administrative role and major responsibilities as an academic nursing leader (ANL).
a. Managing staff, designing and implementing change, engaging in strategic planning and decision‐making, as well as managing educational crises that arose during the pandemic.
4. Did you feel prepared upon taking on the role? How long did it take for you to feel confident/competent?
5. What was the onboarding process when you started this role? Was it formal onboarding, learning on the job, etc.?
6. During the pandemic, describe your perceptions of the greatest challenges to your program.
7. Overall, how did the pandemic influence your educational program’s ability to deliver quality nursing education? For example, program curriculum, student learning and clinical placements, virtual teaching, staff morale, resource allocation, etc.
8. What specific challenges, for example, operational, resources, student/faculty/staff relations, did you experience as the nurse leader?
9. What strategies did you use or develop during the pandemic to facilitate the work of the program and support students, etc.?
10. Describe, if any, the type of leadership style that guided your actions during this period.
11. If possible, describe how your previous leadership experiences may have influenced and benefited your leadership responses during the pandemic.
12. Reflecting on previous leadership experiences and those during the pandemic, describe any new insights, knowledge, or skills regarding crisis leadership.
13. What types of educational innovation(s) did your program or department adopt as a result of this pandemic?
14. If possible, describe any leadership training for academic nursing leaders, responding to future major crises.
15. Is there any additional information you would like to share?

Qualitative data collected from the interviews underwent a rigorous phenomenological analysis, focusing on identifying emergent themes and patterns related to nursing department governance, leadership styles during crises, specific challenges faced, and factors contributing to successful or unsuccessful pivots. The goal was to uncover the essential structures and meanings inherent in the multifaceted role of higher education nursing leadership during the pandemic. An initial review of the data was undertaken to identify potential themes. Further coding was completed, resulting in the final themes, which were reviewed for integrity and coherence.

Data analysis proceeded through an iterative and interpretive process. Interview transcripts were read multiple times to support immersion in the data. Initial coding focused on participants’ descriptions of leadership experiences, including moments of uncertainty, decision‐making, adaptation, relational responsibility, and system‐level disruption. Codes were then compared and grouped into broader categories that reflected recurring patterns across participants’ accounts.

As analysis progressed, the research team engaged in analytic discussions to refine categories and develop themes. These discussions examined whether emerging themes were sufficiently grounded in participant accounts and whether they captured both the practical and meaning‐oriented dimensions of academic nursing leadership during the pandemic. Complexity leadership theory was used as a sensitizing framework during later stages of interpretation in order to examine how leadership emerged within dynamic relationships among academic programs, faculty, students, clinical partners, regulatory expectations, institutional policies, and public health directives.

Themes were, therefore, developed through both inductive attention to the data and interpretive engagement with the study’s theoretical framework. This approach allowed moving beyond description of what leaders did during the pandemic toward analysis of how participants made meaning of leadership in a complex and rapidly changing academic nursing context.

Several strategies were used to enhance the trustworthiness of the study. Credibility was supported through close engagement with interview transcripts, the use of participant quotations, and ongoing analytic dialog between the researchers. Preliminary interpretations were discussed among the research team to examine whether emerging themes were grounded in the data and reflected participants’ accounts of academic nursing leadership during the pandemic.

Dependability was enhanced through documentation of analytic decisions, including coding notes, theme development, and revisions to the thematic structure. Confirmability was supported through reflexive discussion of the researchers’ assumptions, professional locations, and prior experiences in nursing education and academic leadership. As researchers with experience in nursing education, our familiarity with the context provided important interpretive sensitivity while also requiring deliberate reflexivity. We, therefore, returned repeatedly to the transcripts to ensure that interpretations were grounded in participants’ descriptions rather than solely in our own experiences of academic leadership.

Transferability was supported through description of the study context, participant roles, and the Canadian academic nursing education environment during COVID‐19. Although the findings are not intended to be statistically generalizable, the detailed account of participants’ experiences may resonate with readers working in nursing education, health professional education, and academic leadership contexts facing complex crisis conditions.

## 3. Results

Three themes were derived from the combined analysis of the data. These themes included: *(1) Maintaining quality nursing education programs; (2) Administrative decision challenges; and (3) Communication during a crisis*. These themes and subthemes are displayed in Table 2.

**TABLE 2:** Themes and subthemes.

Theme	Subtheme
Maintaining quality nursing education programs	• Transitioning to a technology‐enhanced curriculum • Disruptions to clinical practicums
Administrative decision challenges	• Structural barriers to effective leadership • Jurisdictional and policy differences • Transitioning to the role
Leadership communication during a crisis	• Information sharing • Sustaining collaborative relationships • Empathic communication

### 3.1. Maintaining Quality Nursing Education Programs

#### 3.1.1. Transitioning to a Technology‐Enhanced Curriculum

The rapid move to online teaching reflected more than logistical adaptation. Participants described the experience as “holding together educational continuity, professional standards, faculty capacity, student progression, and clinical learning expectations under conditions of uncertainty.” This statement illustrates that academic nursing leadership during the pandemic involved continuous sensemaking across overlapping educational, regulatory, and health‐system pressures.

As previously reported in the literature, the sudden and rapid change to virtual classroom teaching presented major challenges to all programs in this study [2]. During the initial stage of the pandemic, decisions as to how to prioritize the use of faculty teaching skills and resources to ensure program continuity were the major leadership focus. Disruptions preventing in‐person learning required rapid adaptation to changes in the curriculum. The quality of teaching, especially the need to develop relationships with students, was a common concern: “*The challenge was to help faculty and students to morph into an online sort of thinking, rather than face-to-face. We’ve always done it this way in nursing*. *It takes time to adjust to all this. There’s all this concern about AI. The issue is dehumanization, with all this technology, because asynchronous takes skill to develop rapport with students online”* (P 4).

The need for organizational support during rapid system changes was evident. Participants noted that the availability of campus technology infrastructure and resources varied: *“We needed orientation, faculty needed development on how to run an online class and then manage the technology. At first, they were scared to death. That everything was going to crash and burn”* (P 6). Programs with ready access to resources and support adapted effectively to the complexity of program changes, which is essential to a successful crisis response: *“If there’s ever a silver lining of the pandemic, that would be it. It exposed us to online teaching and, you know, and got us to be cool with it” (P 4).*

Faculty differed in their ability to transition to online teaching; those with little or no prior background experienced more technological challenges and a lack of confidence. Overall, the respondents perceived students being able to adapt to virtual learning more readily since it provided them with more scheduling flexibility and access to additional learning resources. This interpretation was based on leaders’ accounts of faculty workload, rapid course redesign, technological learning curves, and the additional pedagogical labor required to move nursing education online. Faculty may not have recognized the full potential of online teaching since adapting to rapid changes was more difficult in a crisis context.

#### 3.1.2. Disruptions to Clinical Practicums

Clinical practice courses are foundational to nursing programs. Ensuring access to clinical practice sites was more challenging than anticipated. One participant commented: “*The system collapsed on us, we could not have students go to clinical anymore or weren’t allowed to go into any acute settings. But we still had these students who we were trying to get to the point where they were going to be able to stay on top*” (P 12).

Well‐established working relationships with other organizations are a valuable resource during a crisis. Clinical practice partners who supported student learning despite the pandemic lockdowns were essential to program adaptability. These healthcare facilities viewed students as assets since they provided support to the care team. Participants with a strong belief about the positive role of nurses during a global pandemic were essential to sustaining the program. Several expressed the view that: “*Continuing to promote clinical practice learning was consistent with the nursing code of ethics to provide patient care during times of public health crisis. Practice partners welcomed student nurses since they provided extra support for staff*” (P 1).

During periods of organizational disruption, setting priorities and promoting adaptation to sustain operations is a major responsibility of complexity leadership. As documented in the literature, the greatest challenge was to provide all students with the requisite prepandemic clinical practice hours or alternatives for a variety of courses while ensuring student safety [8]. Concerns about equitable opportunities for students as well as sufficient learning experiences to meet competency standards were a common challenge.

Varying degrees of program adaptation were cited. Innovations included developing alternatives such as learning in laboratories or vaccination clinics and prioritizing learning experiences for senior students. Some leaders shifted the emphasis away from required clinical practice hours to focusing on evidence of new learning and skills demonstrated by students. As one participant explained: “*Meeting regulatory requirements is not about the hours of practice completed, but it is the ability of the student to demonstrate the required competencies”* (P 18). This refocusing of educational goals illustrates that effective leaders need to have the ability and knowledge to integrate the key mandate of nursing education programs to meet regulatory requirements.

Historically, alternative ways to teach practice‐based skills with associated safety risks for clients have been used in aviation and medical education. The uptake by nursing began in the 1900s by using mannequins to teach laboratory skills. Simulated learning (SL) using full‐scale computerized patient simulators to present authentic clinical manifestations and clinical scenarios has been viewed as an ideal substitute for traditional clinical practice [19, 20]. Hanshaw and Dickerson [21] concluded that teaching methods which incorporate SL led to increased critical thinking, self‐efficacy, self‐confidence, clinical judgment and motivation (p. 5).

Responding to the pandemic crisis required a rapid transition to technology‐based teaching and learning. Most programs adapted quickly by substituting direct clinical practice in healthcare settings with SL modules. Some faculty praised simulation as a useful tool while others criticized the transferability to real‐world nursing. As noted by participants: *“Many of the faculty apparently did not feel that simulated learning was an adequate replacement for direct clinical practice. They felt that the students were not competent unless they had hands on practice*” (P 17). Faculty acceptance of and adaptation to new innovations, especially during a crisis, is a common challenge that leaders must address [22].

### 3.2. Administrative Decision Challenges

Academic departments and their leaders have a substantial degree of decision‐making autonomy over program operations in noncrisis circumstances. A participant stated: “*That’s the one advantage of being a dean of the faculty of nursing. You are at all the decision tables. You are the direct voice. Nobody’s filtering things”* (P 15). However, during a global pandemic with high mortality rates, the types and pathways for rapid decision‐making became more uncertain and complex. Participants had to balance competing demands and risks while ensuring compliance with academic standards and pandemic precautions. One participant described the leadership experience as: “*Like being a multi-armed octopus,*” often having to make multiple decisions with minimal or conflicting information from different sources (P 20).

#### 3.2.1. Structural Barriers to Effective Leadership

The autonomy to make decisions during crisis conditions is essential in complex organizations. Irrespective of the educational program size, new additional communication pathways and reporting structures between various stakeholders created potential confusion and inefficient timely decision‐making. One participant stated that*, “We have so many people to consult it became difficult to decide who we should listen to”* (P12). Continuously seeking approval to make necessary program changes hindered adaptation. One participant used the analogy*: “If I yell iceberg, I want the Titanic to move. I want that sense of empowerment. I don’t want to be second guessed. I want to be trusted, to have latitude and autonomy that I can drive my own ship”* (P 10). Working in this rapidly changing environment, leaders require the support and trust from more senior administrators to make effective decisions; otherwise, a sense of powerlessness may occur, delaying adaptation.

#### 3.2.2. Jurisdictional and Policy Differences

Obtaining pandemic‐related information from multiple sources and developing alternative pathways and solutions is essential in crisis conditions. Inconsistent policies and directives between different key stakeholders within and external to the university can become a major challenge. A participant shared that: *“Communication came from the university and through other channels from the Health Region. We needed to know exactly what were the rules, the guidelines, the requirements from both. And it got a little frustrating when they were contradictory and not clear”* (P 4).

The lack of system‐wide crisis management strategies delayed adaptation to rapidly changing challenges. Senior university administrators who had little experience or knowledge of health‐related crises were unable to effectively support specialized programs [10]. This created conflicting or ambiguous policies and practices between universities, health councils, health authorities, local, provincial, and federal government, and healthcare sites. Major decisions as to how much and when students would have access to labs, clinical sites, and on‐campus learning created more disruptions. Participants reported that: “*The difficulty was the unpredictability, the constant change. As understandings grew around COVID, things changed constantly, what units we could utilize for students, what staff could go on the units*” (P 2 & P 8).

#### 3.2.3. Transitioning to the Leadership Role

Academic nursing leaders without prior senior administrative experience did not have the opportunity of “learning on the job.” The urgency of adapting to crisis conditions requires immediate leadership action. One participant illustrated the difficulties of inadequate experience in handling major disruptive events: *“One of the most important things where it’s unknown territory and have not experienced the situation before, people react in different ways. Some leaders who are still able to focus on the information they have at hand and make an informed decision with that information” (*P 18). The lack of comprehensive knowledge of operational issues prevents successful adaptation to major program disruptions [12]. A participant reflected the role challenge, stating that: “*It’s difficult to have somebody come in that doesn’t know a program well, it takes so long to build your relationships with people that you need to work with in the various different departments and to develop that rapport, so that you have good working relationships”* (P 9).

The ability to draw on prior experiences or knowledge is foundational to complexity leadership. Academic leaders who were new to their academic role relied on prior leadership lessons learned from the healthcare or business sectors. Some participants had previous experience with a similar major health crisis such as SARS and H1N1. Those with a public health nursing background were also more readily able to adapt with flexibility to the operational challenges. They reported that, *“COVID-19 is similar to other community health problems so just need to apply our knowledge and expertise to caring for patients*” (P 15). Using the opportunities to initiate new innovations by drawing upon collegial networks within their home institution or externally was evident. Access to professional support and shared knowledge was a priority. Many were part of province‐wide networks of nursing leaders, which served as a sounding board for decision‐making, stating that: *“As a new dean making decisions based on my knowledge, judgment, principles and logic, I need to know that I’m consistent with what other people are doing, if I’m missing anything”* (P 7).

### 3.3. Communication During a Crisis

#### 3.3.1. Information Sharing

Creating frequent effective communication opportunities is essential to managing complex organizational disruptions like the pandemic [4, 8, 22]. Ensuring that relevant information was shared in a timely and transparent manner to promote adaptation is a priority for leaders. A participant noted that: *“Policy changes, legislation and the regulations were coming down fast and furious and changing regularly. Working closely with the communications department, you wanted to make sure you got the communications right for the entire college and making sure it was consistent with the message that went out last”* (P 16).

Communication during a crisis takes many forms. Insufficient communication or communication overload were cited: “*It’s an ongoing kind of dilemma on how to communicate and how often, how frequent, what method, what mode?”* (P 12*).* Students needed continuous updates regarding course scheduling and access to clinical practice opportunities. Faculty also required ready access to information in order to make substantial changes to online teaching and testing. One participant shared, “*This is what emergency communication looks like, keep it brief, clear, and send it out as much and trying to work on being transparent, and sharing as much as we could without overwhelming people with information overload. Helping people to be informed so they could stay calm and trying to infuse that confidence that they can do this”* (P 1).

Disruption of routine methods of communication can promote development of new innovations [17]. Programs increased sharing of information using social media apps and video conferencing, which also promoted community building. Participants noted that, *“I had so many announcements from the top down to share, it was taking up too much of our one-hour meeting. I created a newsletter*” (P 6). Participants also reported creating more opportunities for communication: *“We were meeting weekly… getting messaging out to answer questions, even though there was a lot of email communication. Some people were overwhelmed, didn′t understand it in its entirety, and needed clarification. We would have regular meetings weekly, open meetings. What are we talking about? What questions do they have? Just to, kind of, talk through some things” (P 5).*

Facilitation skills to promote team decision‐making are essential to sustaining relationships within the organization [14]. One participant shared, *We had those conversations about the risk for the student? What’s the learning? What does the student need? We balance people’s fears and concerns. I tried to empower faculty. Talk it out with me if you want to. Let’s keep coming together and make these decisions jointly and recognize how we can support each other. Leadership at its best. People thrown together, people learning from each other* (P7).

Supporting team members and promoting collaboration was also a key leadership responsibility. One participant recounted that, *“To make sure that the teams are strong…motivate* them *to move forward with changes to the program*” (P 9). Participants recognized the need to create structured meetings and more informal opportunities to connect with one another. One participant noted that, *“I tried to figure out how can we keep people connected more socially. I try to have open drop-in sessions where people could just come and join me for half an hour. We′d have coffee together”* (P. 5).

#### 3.3.2. Leading Through Empathic Communication

Turnipseed & VandeWaa [13] describe crisis intervention as “taking action to identify and mitigate the human, emotional and physical pain of a crisis” (p. 155). During the pandemic, all participants faced the challenge of supporting faculty, students, and students who were coping with fear, anxiety, uncertainty, and sudden changes to their lives. Recognizing that everyone reacted and coped differently to stressors was critical to successful adaptation. As shared by one participant, “*A result of the rapid changes, of losing their ability to ground themselves in ways that they used to, I see a lot of trauma-related behaviors, and those fears are showing up in relationships. They’re showing up in communication styles*” (P 8). Leaders were highly sensitive to the need to be consistently available and supportive. One participant reflected: “*I think it’s where you are in relation to your staff, your faculty, the people and how you can support them, encourage them. Because that’s ultimately, what’s best for the students. Faculty want to know that they will get support from you*” (P 2).

A strong commitment to the “duty of care” by developing and maintaining trust with faculty, students, and staff is especially important during a crisis. Being compassionate with team members and empathic communication enhances collaborative decision‐making and adaptation to change [6]. As described by another participant: *“We are doing everything to ensure faculty getting the support that they need. Those you are leading are crumbling and need support. We were scared, we weren’t sure what was going on. The students were shaking. There was a softness even in the way we talked with each other. We really had to be good, be kind. We’re struggling on a different thing. We had to really listen. We rallied, we did our best, we did as a leadership team to be very informative”* (P11). (Table 3).

**TABLE 3:** Analytic exemplars.

Theme	Lived experience meaning	Exemplar quote	Interpretive insight	Link to complexity leadership
Maintaining Quality Nursing Education	Leaders had to make continuous decisions regarding how to adapt conventional ways of teaching to new and innovative technology‐based curricula	*“challenge to help faculty and students morph into online thinking”; “the system collapsed, no clinical practice”*	Leaders experienced crisis leadership as holding educational systems together while rules changed rapidly	Adaptive, creative leadership; future focused; organizational sustainability
Administrative Decision Challenges	Leaders had to adapt to multilayered barriers and challenges to ensure rapid responses to the ongoing crisis	*“pandemic unknown territory; people adapt differently;* *need to use information to make informed decisions.*	Crisis decision‐making involves managing multiple risks to promote adaptation and innovation	Complexity leadership focuses on addressing competing demands and priorities
Communicating During a Crisis	Leaders engaged in continuous communication to ensure transparency and a sense of cohesion	*“policy and information changes were fast and furious; get messages out to help people to stay calm and confident”*	Disseminating essential information in a timely manner as pandemic circumstances change is a major leadership role	Updating all members of the organization and promoting collaboration are key strategies of complexity leadership

## 4. Discussion

Decision‐making during the recent pandemic illustrated that adhering to familiar routines no longer applied. Leadership required during periods of organizational stability is significantly different in crisis conditions [9]. Senior leaders need to be able to make critical decisions to minimize disruptions while sustaining an optimal level of operation [12, 13]. This type of decision‐making was often difficult since there were many unknown factors, safety issues, and a lack of a clear right or wrong path forward. Leadership strategies need to be modified during the ongoing stages of a pandemic. Uhl‐Bien et al. [11] argued that healthcare or similar organizations responding to major crises with traditional bureaucratic leadership will be unsuccessful. Doing more of the same would be ineffective in light of the scope and depth of the changes created by a pandemic [3].

Instead, responding from a complexity leadership lens recognizes the need for collaboration to enhance adaptability and fitness of the system. This approach to leadership not only sustains the organization during the crisis but also promotes innovation, thereby increasing its future adaptability [11]. Ajith et al. [3] recommend an adaptive crisis management focusing on sustaining operational levels of the organization. During the early stages of the pandemic, the major role of nurse leaders would be to assist all team members in identifying adaptive outcomes and continuous improvements. This type of adaptation leads to new innovations rather than maintaining the status quo, but it is often not viewed as a priority [9]. The successful nurse leaders in this study were able to navigate unexpected barriers, which was critical to mitigating the negative impact of the crisis.

A common theme was the lack of preparedness in responding to the magnitude of the challenges associated with a global crisis. As found in previous studies, pre‐existing educational program issues became more apparent with the pandemic highlighting the complexity of delivering nursing education [8]. Changing long‐established operational practices and routines created multiple levels of distress within the organization. Without system‐wide emergency plans, managing risks and disruptions associated with protecting the overall safety of students, faculty, and staff was a major concern [16]. The lack of prior crisis experience increased financial costs associated with integrating SL, faculty development, and effective technical support, creating barriers to the effective use of resources [2, 20].

The major leadership focus in mitigating crisis situations is to rapidly create a coordinated system of response to threats and to establish a new level of stability. Instead of working in silos, a governance system that aligns communication and responsibilities is essential [9, 10]. This study provides insight into leaders’ varying ability to successfully recognize and manage the complexities of the crisis by taking actions needed to develop new ways of program delivery. Higher education organizations do not routinely face sudden and major crises and usually are relatively slow in developing new educational practices. But the pandemic created situations whereby leaders had to suddenly learn crisis management and crisis intervention strategies [2, 13]. Some recognized that “*the pandemic forced us to be different in a way that we could open our minds”* (P 19). Actions that significantly restrict negative consequences while providing the necessary support to minimize physical and mental distress became a priority during a crisis.

A common and yet unexpected program challenge, reported in the literature and also evident in this study, was the sudden transition to an entirely different mode of program delivery in a short period of time. Online learning and virtual classrooms are well‐established in nursing education programs [20]. Online and blended learning had become increasingly established in Canadian higher education prior to the pandemic; however, the rapid and large‐scale shift required during COVID‐19 was unprecedented in scope and pace for many nursing programs.

This technological advancement provides a greater ease of access for students and provides a wider variety of programs. During the pandemic, however, all universities had to transition to remote, technology‐enhanced teaching for an undefined period of time, creating major operational challenges. Decision‐making during the early stages of the pandemic required leaders to consider how to best utilize existing resources and prioritize student learning needs, balancing this with faculty expertise and willingness to adapt to new methods of teaching. However, the results illustrated that Canadian undergraduate nursing programs varied in their ability to effectively offer online curricula.

The degree of adaptability is largely dependent on faculty having prior experience and success with online teaching. Programs with leaders and faculty who had the knowledge and ability to support others were most successful in promoting rapid adaptation to new teaching skills. It was not simply maintaining the prepandemic quality of teaching, but rather, the goal was to integrate new and innovative approaches to quality learning. Overall, students adapted quickly to virtual learning for in‐person classroom courses, identifying key benefits such as reducing the need to travel, having greater access to course materials, and experiencing less exposure to high‐risk contacts. An unexpected but positive outcome was the difficulty of reverting back to in‐person classrooms later because students preferred the benefits of virtual learning.

The sudden change to virtual teaching had the most dramatic impact on identifying effective substitutes for clinical practice courses either on campus or in healthcare settings [5]. Limited access to sufficient clinical practice opportunities has been a long‐standing issue for many undergraduate health professions programs. Again, this highlights the fact that often during crisis conditions, issues that have not been addressed create significant problems. The current literature supports the benefits of SL as a major component of clinical practice education. Yet, in this study, there was either a reluctance to integrate this learning resource or a lack of clarity regarding its benefits. Hanshaw and Dickerson [21] concluded from a review of current SL practices that the development of more concise operational definitions of clinical competence in nursing practice is needed in order to fully evaluate the effectiveness of SL. Several areas that warrant further research include exploring the extent to which students’ increased confidence, knowledge, and expertise are effectively transferred to clinical practice environments [19]. Technology should enhance the ability to provide quality education, not the reverse.

In unstable environments with intense role demands, successful nurse leaders require the ability to sustain their educational programs. Despite similarities between programs, an unexpected result was the different types of leadership behaviors exhibited by the program leaders. Positive leadership characteristics are vital to managing the complexities of crises, yet they have received less attention in the postpandemic academic literature. Fowler & Robbins [18] concluded, following a study of registered nurses, that a transformational style of leadership was most effective during the pandemic. These characteristics represent a multidimensional, goal‐directed approach to crisis management. Role modeling positive behaviors provides incentives for staff adaptation. A clear and transparent style of communication fosters contributions from the collective group. Recognizing the personal interests and safety of individual staff conveys a positive regard for their needs. Consistent with the goals of complexity leadership and crisis adaptation, fostering intellectual stimulation to assist organizational problem solving and implementing innovations is needed.

The adaptability and resilience of the leaders in this study suggest that the transformational style of leadership would be applicable to other nursing education contexts. In addition, to responding to the needs of the students and faculty, they used their pre‐existing knowledge and well‐established expertise to instill confidence and ensure collective problem‐solving. Some leaders recognized the high degree of distress and disruptions to faculty, staff, and student lives and established ways to ameliorate potential harms. They provided consistent support to all members of the programs, communicating a vision and plan of action for collective goals to sustain program operations. These types of leadership skills are most likely to promote organizational innovation and adaptation [23].

Studies of leadership during the pandemic illustrated that effective leadership is an adaptive process rather than an identifiable set of traits or behaviors [3]**.** While it is important to acknowledge that certain types of leadership styles were identified, during crisis conditions, successful leaders are able to manage the complexities of their organization by setting priorities and making critical decisions. They were agents of change, promoting innovativeness and resilience. Leaders who recognized the interconnections within the faculty and the broader institutions and the unpredictability of the issues and events evidenced a deep understanding of complex adaptive systems.

Complexity leadership skills, however, develop over time [24]. Those who lack these skills could hinder problem‐solving and innovation, especially during a crisis [10, 18]. The results illustrated that nurse leaders with the least experience had major difficulties in this role. An in‐depth understanding of the operational aspects of the nursing program and its interconnection with the larger university and external healthcare system was essential [3]. The combined knowledge and skill deficits hindered their ability to address the complexity of the issues for the duration of the crisis. The ability to demonstrate a key component of complexity leadership, namely, initiating successful problem‐solving and new innovations in a timely manner, was not evident.

The least experienced leaders lacked the ability and confidence to prioritize actions to achieve the most benefits in overcoming a crisis and most likely were not receiving adequate support. Converting in‐person teaching to completely virtual methods within days or weeks presented insurmountable challenges. In some cases, faculty resistance to essential program changes or the lack of resources created feelings of self‐doubt, anxiety, and disillusionment about the role. They experienced similar types of traumatic effects as nurses on the frontline [25]. The overall result was a lack of success in mitigating the negative effects of the pandemic crisis on the program.

Canadian university operations have become more complex [10]. Program leaders, even during more stable periods, must be competent in labor relations, financial and human resource management, as well as collaborating with government, professional associations, and regulatory bodies. It is, therefore, not surprising that inexperienced academic nursing leaders experienced major role difficulties during a global health crisis.

Recommendations for organizations to better prepare for similar types of crises in higher education were identified by the participants. Most universities lacked pre‐existing emergency plans to respond to the pandemic, which resulted in conflicting decision‐making and not receiving vital information in a timely manner [16]. More attention needs to be directed to emergency planning that anticipates the unique programmatic needs of nursing and other professional practice programs [4, 11]. This was recognized by one participant who acknowledged that: “*If a pandemic happens again, what are the lessons learned across Canada in schools of nursing or globally to adapt? We need to learn from each other. That would be one of the takeaways and educate our leaders, so that they can be better prepared*” (P11).

Fowler & Robbins [18] emphasize the importance of continuous support at all levels of an organization during a crisis. All participants identified the lack of consistent and coherent guidance from senior academic administrators, especially during the early phases of the pandemic. Increased policy directives, while clarifying major priorities, were needed. Inadequate leadership by senior administrators during crises impacts all programs and the overall organization in the short and long term. Disruptions to collegial working relationships can ultimately damage the morale and productivity of faculty and the university [10].

During a major health crisis, many academic nursing leaders can contribute within the university and beyond. As evidenced in this study, many of the academic nursing leaders would have benefited from greater respect for their knowledge and expertise from senior administration. They required increased autonomy in decision‐making regarding the unique needs of their programs without facing multiple levels of authorization. Several participants actively contributed to the universities’ pandemic response, but the majority had minimal input regarding vaccine policies, resource distribution, staging the return to in‐person teaching and learning, and postpandemic recovery. Wignall & Mason [26] assert that the nursing profession needs to enhance its engagement with healthcare policy, especially with a focus on health promotion and safety issues. Significant contributions by nursing education leaders to emergency planning, regulatory and provincial funding policies in nursing and higher education are needed [27].

Moerschell & Novak [9] recommend implementing holistic crisis management systems that address planning, response, and recovery in universities. A similar goal is recommended for academic nursing leaders in Canada. This study revealed a wide range of abilities and responses to pandemic challenges during a major crisis. Establishing collaborations between local, provincial, and national nursing education/practice networks would have created a more cohesive and adaptive approach to pandemic challenges. It would have provided ready access to additional resources and mentoring by senior leaders for less experienced leaders.

This study contributes to nursing leadership scholarship by illuminating the often‐invisible work of academic nursing leaders during a crisis. Unlike clinical nursing leaders, who were often positioned at the point of direct care delivery, academic nursing leaders navigated the intersection of educational continuity, regulatory requirements, student progression, faculty workload, clinical placement disruption, institutional policy, and public health directives. Their leadership work involved maintaining the integrity of nursing education while responding to uncertainty across both higher education and health‐system contexts. This distinction is important because academic nursing leadership requires preparation not only in educational administration but also in crisis sensemaking, relational coordination, ethical decision‐making, digital pedagogical adaptation, and cross‐sector collaboration.

In order to be prepared for potential future crises, a major implication from this study is the need for nursing education programs to engage in succession planning. Participants’ leadership experiences contributed to a deeper, nuanced understanding of the knowledge and skills required by future leaders. Leadership succession planning consists of “a systematic, strategic effort to ensure leadership transition into critical positions and essential to preventing a leadership vacuum, loss of institutional knowledge, morale and engagement issues from faculty and staff, which hinders innovation” ([28], p. 323). Leadership development and mentoring for those who show potential are also required to maintain organizations’ long‐term success [15, 24, 29].

Further research is needed to identify the key components of complexity leadership development. The primary focus proposed by Al‐Dabbagh [12] consists of training potential candidates in critical and creative thinking skills that would be most needed during the various stages of an organizational crisis. Dopson et al. [30] suggest that social capital must be included by building networked relationships among multiple individuals in order to enhance organizational effectiveness. The objective is to enhance leaders’ decision‐making ability to systematically identify alternative actions as rapidly as possible while avoiding negative outcomes. Cummings et al. [6] identified major components of nursing leadership development programs. These programs need to be evidence‐ and theory‐based, adapted to the learning needs of the participants, provide ongoing mentoring, and foster a trusting relationship between mentor and mentee. Clearly, there is no generalized approach to leadership development. The most successful are those with a targeted approach to the specific needs of the organizational context.

## 5. Limitations

This study contributes to the additional understanding of a pandemic‐induced crisis affecting nursing education leadership, which has received little attention. The results represent the views of a mixed group of senior nurse administrators whose role responsibilities varied. Some held more senior positions than others, but the only focus of the study was on their experiences with the undergraduate programs. Some variability in the findings is expected due to different levels of knowledge and types of administrative experiences, the size and geographical location of the programs, and the composition of faculty and staff. The transferability of the results should be considered in the context of the type of curriculum in Canadian undergraduate nursing programs.

## 6. Conclusion

Gigliotti [31] asserts that especially during the chaos and dark times of a major crisis, effective leadership is vital to ensuring successful adaptation at all levels of an organization. Examining Canadian academic nursing leaders’ lived experiences provided the opportunity to identify the necessary knowledge and skills that promote or hinder complexity leadership, and how these qualities interact with situational factors to predict positive or negative outcomes for stakeholders. Their contributions, while valuable, have been less visible within the overall healthcare system. Nevertheless, they demonstrated courage, resilience, and deep caring for all those for whom they were responsible.

The pandemic offered opportunities for learning how to adapt to major disruptions and develop new innovations and organizational growth [1, 2, 11, 17]. Nursing education programs were not immune to disruptions, as witnessed by the dramatic short‐ and long‐term changes caused by the pandemic. With the potential for the occurrence of future crises, attention to enhancing the adaptability of the overall university and its programs to address the complexity of the challenges is recommended. Preparing future leaders through succession planning is a vital resource. Barling [24, 32] recommends that further research is needed to improve leadership selection, training, and development in the event of future crises.

## Author Contributions

Lillie Lum: conceptualization, funding acquisition, methodology, investigation, participant recruitment; data curation, writing–original draft preparation, review and editing of all drafts, and project administration. Liza L. S. Choi: contributed to conceptualization, methodology, investigation, participant recruitment, funding acquisition, project administration, data interpretation, substantial manuscript revision, writing–review and editing, and final manuscript preparation.

## Funding

This research was funded by York University and Mount Royal University.

## Conflicts of Interest

The authors declare no conflicts of interest.

## Data Availability

Data can be made available by contacting Lillie Lum, York University, Toronto, Canada: email lum@yorku.ca.

## References

[1] Caralon C. , Davies Cl. , Crookes P. , McGhee S. , and Roxburgh M. , COVID-19: Disruptive Impacts and Transformative Opportunities in Undergraduate Nurse Education, Nurse Education in Practice. (2020) 46, 10.1016/j.nepr.2020.102807. PMC7255089 32502757

[2] Williamson K. , Nininger J. , Dolan S. , Everett D. , and Joseph-Kemplin M. , Opportunities in Chaos Leveraging Innovation to Create a New Reality in Nursing Education, Nursing Administrative Quarterly. (2021) 45, no. 2, 159–168, 10.1097/NAQ.0000000000000464. 33570884

[3] Ajith N. , Lux A. , Bentley T. , and Striepe M. , Adaptive Crisis Management at the Operational Level: Responses to COVID in the Australian Resources Sector, Journal of Management and Organization. (2025) 31, no. 4, 1909–1933, 10.1017/jmo.2024.62.

[4] Fara S. , Smith S. , Qureshi K. , and Stalter A. , Nursing Education Leaders Decision-Making During a Pandemic Crisis, Nursing Education Perspectives. (2022) 43, no. 5, 297–299, 10.1097/01.NEP.0000000000000889. 36037419

[5] Dewart G. , Corcoran L. , Thirsk L. , and Petrovic K. , Nursing Education in a Pandemic: Academic Challenges in Response to COVID-19, Nurse Education Today. (2020) 92, May 28:92 10.1016/j.nedt.2020.104471. PMC7263267 32502723

[6] Cummings G. G. , Tate S. L. , Penconek T. , Micaroni S. , Paananen T. , and Chatterjee G. , The Essentials of Nursing Leadership: A Systematic Review of Factors and Educational Interventions Influencing Nursing Leadership, International Journal of Nursing Studies. (2021) 115, 1–11, 10.1016/j.ijnurstu.2020.103842. 33383271

[7] Farsi Z. , Afagh E. , Forniere A. , Ahnadi Y. , and Sajadi S. , Investigating Nursing Students’ Satisfaction With Quality of Courses and Virtual Learning During the COVID-19 Pandemic, Turkish Online Journal of Distance Education. (2021) 23, no. 1.

[8] Ion R. , Craswell A. , Hughes L. et al., International Nursing Education Leaders’ Experience of Responding to the COVID-19 Pandemic: A Qualitative Study, Advanced Journal of Nursing. (2021) 00, 1–9, 10.1111/jan.14892. PMC8242510 34105829

[9] Moerschell L. and Novak S. S. , Managing Crisis in a University Setting: The Challenge of Alignment, Journal of Contingencies and Crisis Management. (2020) 28, no. 1, 30–40, 10.1111/1468-5973.12266.

[10] Mandzuk D. , On the Edge of Chaos: How the Deans at One Canadian University Have Managed to Lead in the Face of the Pandemic and Other Sources of Uncertainty and Complexity, Journal of Higher Education Policy and Management. (2023) 46, no. 1, 18–31, 10.1080/1360080X.2023.2238335.

[11] Uhl-Bien M. , Meyer D. , and Smith J. , Complexity Leadership in the Nursing Context, Nursing Administration Quarterly. (2020) 44, no. 2, 109–116, 10.1097/NAQ.0000000000000407. 32134869

[12] Al-Dabbagh Z. S. , The Role of Decision-maker in Crisis Management: A Qualitative Study Using Grounded Theory (COVID-19 Pandemic Crisis as A Model, Journal of Public Affairs. (2020) 20, 10.1002/pa.2186. PMC7323116 32837321

[13] Turnipseed D. L. and VandeWaa E. A. , Crisis Leadership During and After the COVID Pandemic, The Journal of Nursing Administration. (2022) 52, no. 3, 154–159, 10.1097/NNA.0000000000001123. 35170579

[14] Uhl-Bien M. , Complexity Leadership and Followership: Changed Leadership in a Changed World, Journal of Change Management: Reframing Leadership and Organizational Practice. (2021) 21, no. 2, 144–162, 10.1080/14697017.2021.191749.

[15] Lum L. and Choi L. L. S. , Conrad D. and Archer W. , Chapter 4: the Baffling World of Middle Management in Academia, Portraits of Academic Life in Higher Education, 2025, Brill, Leiden/Boston, 60–74, 10.1163/9789004716117_00.

[16] Weaver K. , Rabbitt M. , Summers S. W. , Phillips R. , Hottenstein K. , and Cole J. M. , Acute Crisis Leadership in Higher Education Lessons from the Pandemic, 2023, Taylor & Francis Group, New York.

[17] Uhl-Bien M. , Complexity and COVID-19: Leadership and Followership in a Complex World, Journal of Change Management Studies. (2021) 58, no. 5, 400–1400, 10.1111/loms.12696.

[18] Fowler K. and Robbins L. R. , The Impact of COVID-19 on Nursing Leadership Characteristics, Worldviews on Evidence-Based Nursing. (2022) 19, no. 4, 306–315, 10.1111/wvn.12597. PMC9349434 35833661

[19] Au M. L. , Tong L. K. , Li Y. Y. , Ng W. I. , and Wang S. C. , Impact of Scenario Validity and Group Size on Learning Outcomes in High-fidelity Simulation: A Systematic Review and Meta-analysis, Nursing Education Today. (2020) 111, 1–13, 10.1016/j.nedt.2022.105705. 36599250

[20] Li Y. Y. , Au M. L. , Tong L. K. , Ng W. I. , and Wang S. C. , High-Fidelity Simulation in Undergraduate Nursing Education: A Meta-Analysis, Nurse Education Today. (2022) 111, 10.1016/j.nedt.2022.105291. 35158134

[21] Hanshaw S. and Dickerson S. , High Fidelity Simulation Evaluation Studies in Nursing Education: A Review of the Literature, Nurse Education in Practice. (2020) 46, 10.1016/j.nepr.2020.102818. 32623148

[22] Simonson N. , Brown K. , and Klingbeil C. , Academic Nursing Leadership: Lessons Learned during a Pandemic: A Qualitative Research Study, Nurse Education Today. (2023) 120, 1–6, 10.1016/j.nedt.2022.105620. 36403497

[23] Krouse A. M. , McLaughlin K. M. , Rossi R. et al., Nurse Faculty Administrators’ Experiences of Communication, Cooperation, Coordination, and Collaboration During Educational Disruption, Nursing Education Perspectives. (2022) 1, 1–5, 10.1097/01.NEP.0000000000001013. 35853130

[24] Barling J. , The Science of Leadership: Lessons from Research of Organizational Leaders, 2014, Oxford, New York.

[25] Lum L. , Covid-19 and the New Normal of Mental Health Challenges for Nurses at the Frontline, Journal of Concurrent Disorders. (2020) 3, no. 1, 1–13, 10.54127/UWYS7639.

[26] Wignall A. and Mason D. J. , Mandate from the Pandemic: Nurses as Policy Leaders, International Nursing Review. (2022) 69, 1–3, 10.1111/inr.12742. 34988988

[27] Kagan I. and Hirshfeld M. J. , Nursing Leadership in Drawing Policy Lessons from Major Events Such as a COVID-19 Pandemic, Journal of Nursing Scholarship. (2023) 55, no. 1, 5–7, 10.1111/jnu.12865. PMC9878144 36494839

[28] Schweitzer K. and Graebe J. , Advancing Nursing Practice: The Evolution and Impact of Continuing Professional Development, The Journal of Continuing Education in Nursing. (2024) 55, no. 4, 153–156, 10.3928/00220124-20240318-0. 38551508

[29] Ting J. J. and Babenko-Mould Y. , Early Career Nurses’ Experiences of Engaging in a Leadership Role in Hospital Settings, Canadian Journal of Nursing Research. (2024) 56, no. 3, 257–268, 10.1177/0844562124/24/236666. PMC11308292 38449305

[30] Dopson S. , Ferlie E. , McGivern G. et al., Leadership in Higher Education: a Literature Review and Implications for Programme Design, Higher Education Quarterly. (2019) 73, no. 2, 218–234, 10.1111/hequ.12194.

[31] Gigliotti R. A. , Looking Beyond COVID‐19: Crisis Leadership Implications for Chairs, The Department Chair. (2019) 1, no. 31, 14–15, 10.1002/dch.30330. PMC7361460 40477103

[32] Maaka T. , Pless N. M. , and Wohlgezogen F. , The Fault Lines of Leadership: Lessons from the Global Covid-19 Crisis, Journal of Change Management: Reframing Leadership and Organizational Practice. (2021) 21, no. 1, 66–86, 10.1080/14697017.2021.1861724.

